# Prevalence of antenatal depression and associated factors among pregnant women in Addis Ababa, Ethiopia: a cross-sectional study

**DOI:** 10.1186/s12978-015-0092-x

**Published:** 2015-10-30

**Authors:** Abera Biratu, Demewoz Haile

**Affiliations:** Department of Nursing, College of Medicine and Health sciences, Madwalabu University, Bale Goba, Ethiopia; Department of Reproductive Health, College of Medicine and Health sciences, Bahir Dar University, P.Box 79, Bahir Dar, Ethiopia

**Keywords:** Antenatal care, Depression, Maternal health, Mental health, Low income countries, Ethiopia

## Abstract

**Background:**

The World Health Organization identifies depressive disorders as the second leading cause of global disease burden by 2020. However, there is a paucity of studies which examined the associated factors of antenatal depression in low-income countries. This study aimed to determine the prevalence of antenatal depression and associated factors among pregnant women in Addis Ababa, Ethiopia.

**Methods:**

A cross-sectional study was employed among 393 pregnant women attending antenatal care service in Addis Ababa public health centers, Ethiopia from April 12–26, 2012. The Edinburgh Postnatal Depression Scale (EPDS) was used to detect depressive symptoms. Descriptive statistics and logistic regression were used in the statistical analysis.

**Results:**

Prevalence of antenatal depression was 24.94 % (95 % CI: 20.85–29.30 %). In the final multivariable model, those pregnant women who have previous history of depression were nearly three times at higher odds of having antenatal depression as compared to pregnant women who have no history of depression [AOR = 2.57(95 % CI: 1.48–4.48 )]. Those pregnant women having unplanned pregnancy were nearly three times at higher odds to develop depression as compared to pregnant women whose pregnancy was planned [AOR = 2.78(95 % CI: 1.59–4.85)]. The odd of developing antenatal depression was 89 % higher in those pregnant women who experienced lack of baby’s father support [AOR = 1.89(95 % CI: 1.06–3.36)]. Education level, community’s support, and partner’s feeling on current pregnancy were not significantly associated factors with antenatal depression in the final multivariable model.

**Conclusion:**

Although clinical confirmation for antenatal depression is not conducted, one quarter of the pregnant women attending antenatal care were depressed in Addis Ababa based on EPDS. Unplanned pregnancy, experiencing lack of baby’s father support and previous history of depression were factors independently associated with antenatal depression. Promotion of family planning and integration of mental health service with existing maternal health care as well as strengthening the referral system among public health centers were the recalled interventions to prevent antenatal depression in Addis Ababa Public Health Centers.

## Introduction

Depression is among the most prevalent psychiatric disorders affecting women [1, 2]. Depressive disorders are predicted to be the second leading cause of global disability burden by 2020 [2]. The risk of depression increased significantly during pregnancy [3, 4] and clinically significant depressive symptoms are common in mid and late trimesters [4]. Several studies have reported that depressive symptoms are more frequent during pregnancy than during the postpartum period [3, 5–7].

A meta-analysis study done in the developed countries showed that the prevalence rates of depression (95 % confidence interval, CIs) were 7.4 % (2.2–12.6 %), 12.8 % (10.7–14.8 %), and 12.0 % (7.4–16.7 %) for the first, second, and third trimesters, respectively [7]. A systematic review from low- and lower-middle-income countries revealed that the mean weighted prevalence of antenatal common mental disorders was 15.6 % (95 % CI: 15.4–15.9 %) [8]. High prevalence of antenatal depression has been reported from developing countries i.e. 29 % in Bangladesh [9], 25 % in Pakistan [10], 20.2 % in Brazil [11], 39 % in South Africa Cape Town [12], 38.5 % in South Africa KwaZulu-Natal [13] and 39.5 %, in Tanzania [14].

Depressive symptoms during pregnancy may have devastating consequences, not only for the women, but also for the child and family [15]. Depression during pregnancy negatively influences social adjustment and marital relationships [16, 17]. It affects also the mother‐infant interaction through its influences on the occurrence of postnatal depression [18, 19]. Studies also showed that there is a significant associations between antenatal depression and poor infant outcomes (low birth weight, preterm delivery or both, fetal growth restriction) in low [20–22] and middle‐income countries [23].

Pregnant women with depression are also more likely to suffer from obstetrical complications such as pre-eclampsia [24–26]. Despite the fact that antenatal depression is a common condition with serious consequences, only a few studies have been conducted in low-income countries like Ethiopia. The first study was a validation study of Edinburgh Postnatal Depression Scale (EDPS) on perinatal mental disorder among rural women. That study found a limited validity of EDPS among rural women [27]. However this tool was again tried among postnatal women in urban residents which showed a good reliability and validity [28]. A more recent study conducted on Southwestern Ethiopia found that the prevalence of depressive symptoms during pregnancy was 19.9 % (95 % CI, 16.8–23.1), using EPDS cut off point of 13 and above [29]. The small number of studies conducted in Ethiopia showed that there is a paucity of evidences on antenatal depression to design appropriate intervention. Thus this study aimed to determine the prevalence of antenatal depression and identify factors associated with antenatal depression in Addis Ababa, Ethiopia.

## Methods

### Study setting and design

This study was conducted in Addis Ababa which is the capital city of Ethiopia. The population of Addis Ababa was 2,738,248 in 2007, of which 52.4 % are females, and women between 15 and 49 years of age constitute 34.6 % [30]. There were 26 health centers which are eligible for this study. Maternal health services are free of charge in Ethiopia at Public health institutions including those Public Health centers included in this study. Antenatal care is provided in all public health centers of Addis Ababa without any cost like other maternal health services. An institutional based cross sectional study design was employed in six selected public health centers in Addis Ababa. The data collection period was from April 12–26, 2012.

### Sample size and sampling procedure

The sample size of 422 was determined based on the formula for a single population proportion by assuming a prevalence of antenatal depression of 50 %, confidence level 95 %, margin of error 5 and 10 % for non-response rate. Six public health centers, namely Addis Ketema, Bole, Gulele, Kotebe, Meshualekia, and Yeka, were selected randomly from the 26 health centers that are under the Addis Ababa Bureau of Health. All consenting women attending for antenatal care during the study period in each health center were taken into the study until the sample size was reached. Any apparently healthy pregnant women at any age of gestation who had come to those health institutions during the study period were included in the study. However pregnant women who came for the treatment of any medical diseases including trauma related illness were excluded from the study by assuming that the medical illness they were experiencing during the data collection might affect their mental status. Pregnant women who unable to hear or speak were also excluded from the study. Pregnant women with clinically identified HIV infection, diabetes mellitus, and tuberculosis who were in care during the time of data collection were excluded from the study.

### Measurements

**Fig. 1:**
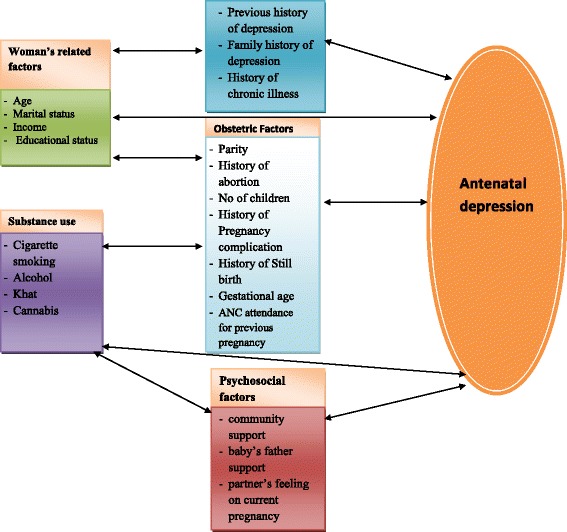
Conceptual framework of antenatal depression and associated factors

The Edinburgh Postnatal Depression Scale (EPDS) [31] has been used to detect depressive symptoms. The EPDS is a 10 item questionnaire, scored from 0 up to 3 (higher score indicating more depressive symptoms), that has been validated for detecting depression in ante partum and postpartum samples in many countries. The instrument was validated in public health centers in Addis Ababa for postpartum use and showed sensitivity of 84.6 % and specificity of 77.0 % at the cutoff score 7/8 [28]. The cutoff point of EDPS among pregnant women is usually higher than postpartum women [32]. Like other similar studies conducted abroad and in Ethiopia, we used EPDS cutoff point of 13 to identify pregnant women with depressive symptom [29, 33]. Those pregnant women who scored 13 and above were categorized as depressed women while pregnant women who scored below 13 were considered as non depressed women [29]. Partner’s feeling on current pregnancy can be defined as pregnant women feeling about the feeling of her partners regarding the current pregnancy. It was measured by asking the pregnant women to rate whether her partner feels happy or not happy on the current pregnancy. Baby’s father support was measured by asking the pregnant women feeling about the partners support to the health of the fetus and continuation of the pregnancy. Community support is measured by asking the pregnant women feeling about the emotional support of the community. The explanatory variables: baby’s father support (poor vs good), partner’s feeling on current pregnancy (happy vs unhappy), community support (poor vs good), and substance use history (yes vs no) were collected by a structured questionnaire. Socio-demographic characteristics and obstetric variables: trimester (first, second and third), having previous pregnancy (yes vs no), previous pregnancy & labor complication (yes vs no), previous history of stillbirth (yes vs no) , previous history of abortion (yes vs no), is the current pregnancy planned (yes vs no), previous ANC follow up (no follow up , sometimes, regular) and current pregnancy complication (yes vs no) were also collected by a structured questionnaire. The conceptual framework is presented in Fig. 1.

### Data collection tool and process

The same Amharic version of Edinburgh Postnatal Depression Scale which has ten items was used as the previous validation study [27]. The questionnaire was prepared first in English then translated in to Amharic and back to English to check for consistency by two independent bilingual Ethiopian final years mental health postgraduate students. The Amharic version of this questionnaire was also used to collect the data. The questionnaire was pretested among pregnant women attending ANC clinic at Arada Health Center. Data were collected by trained, female and experienced diploma nurse holders with a close supervision of the principal investigator and trained supervisors. Two days training was given to the data collectors on the data collection tool, interview technique, eligible study subjects, sampling techniques and consent.

### Statistical analysis

The coded data was checked, cleaned by entering into Epi Info and exported into Statistical Package for the Social Sciences (SPSS window version 16, Chicago Illinois) for analysis. Descriptive statistics was employed to estimate the prevalence of antenatal depression. Bivariate analyses (binary logistic regression) were carried out between the predictors and antenatal depression. Using significant variables (*P* <0.05) from binary logistic regression models, a multivariable logistic regression model was fitted to identify the independent predictors of antenatal depression. The strength of association was measured by odds ratios with 95 % confidence intervals. All tests were two sided and statistical significance was declared at *P* <0.05.

### Ethical consideration

Ethical clearance was obtained from Institutional Review Board (IRB) of University of Gondar. Letter of permission was obtained from Addis Ababa Health Bureau. All Antenatal clinic attending women were approached and participants who gave written consent were interviewed. All the study participants were with age greater than 18 years. The interview was conducted in a separate room in the antenatal clinic. Personal identifying details were not recorded. Participants identified with depressive symptoms were advised to visit the psychiatric clinic for better evaluation and treatment.

## Results

### Socio-demographic characteristics

The response rate for this study was 93.13 %. The mean age of the respondent was 25.27 years (Standard Deviation, SD = 4.335). The age of the respondents ranges from 18–38 years. Three hundred fifty four (90.1 %) of the women were married. Almost 337 (85.7 %) of the women had attended formal education. Majority of the respondents were orthodox by religion 279 (71 %) and Amhara by ethnic group 195 (49.6 %). Majority of the respondents 277 (70.5 %) had a monthly income of above 500 Ethiopian birr (ETB) (Table 1).

**Table 1:** Socio-demographic characteristics of pregnant women’s attending antenatal care at public health centers in Addis Ababa, 2012 (*n* = 393)

	Frequency	Percentage (%)
Maternal age		
< 20 Years	34	8.7
20 – 24 Years	138	35.1
25 – 29 Years	146	37.2
30 – 34 Years	62	15.8
>34 Years	13	3.3
Educational status		
No formal education	56	14.2
Elementary school	166	42.2
Secondary school	116	29.5
Above secondary school	55	14
Religion		
Orthodox	279	71.0
Muslim	85	21.6
Protestant	29	7.4
Ethnicity		
Amhara	195	49.6
Oromo	82	20.9
Gurage	100	25.4
Tigre	16	4.1
Occupational status		
Government employee	22	5.6
Private employee	106	27.0
Running personal business	60	15.3
House wife	189	48.1
Jobless	16	4.1
Monthly income (Ethiopian birr^a^)		
<= 500.00 Birr	116	29.5
>500.00 Birr	277	70.5

^a^1 USD = 20.05ETB

### Obstetric and clinical characteristics

Most of the pregnant women (366; 93.9 %) were in either their second or third trimester at the time of the study. Near to half of the pregnant women (191; 48.6 %) were primigravidas. One tenth of the pregnant women had a previous obstetric complication and 11 (2.8 %) of the women reported history of still birth. Almost one fifth of the women (71; 18.1 %) had experienced abortion. Near to two thirds (248; 63.1 %) of the respondents reported that their pregnancy was planned (Table 2).

**Table 2:** Obstetric and clinical characteristics of pregnant women’s attending Public health centers ANC clinic in Addis Ababa, April, 2012 (*n* = 393)

Explanatory variables	Categories	Frequency	Percentage
Trimester	First trimester	27	6.1
	Second trimester	132	34.4
	Third trimester	234	59.5
Having previous pregnancy	No	191	48.6
	Yes	202	51.4
Previous pregnancy & labor complication	No	352	89.6
	Yes	41	10.4
Previous number of children	0	239	60.8
	1	101	25.7
	2	40	10.2
	≥3	15	3.4
Previous history of still birth	Yes	11	2.8
	No	382	97.2
Previous history of abortion	No	321	81.9
	Yes	72	18.1
Type of abortion	Spontaneous	37	48.61
	Induced	35	51.39
Is the current pregnancy planned	No	145	36.9
	Yes	248	63.1
Previous ANC follow up	No follow up	134	34.1
	Sometimes	10	2.5
	Regular	58	14.0
Current pregnancy complication	Yes	24	6.1
	No	369	93.9
History of chronic illness			
	Yes	367	93.4
	No	26	6.6

### Psychiatric history and substance use

In considering the psychiatric characteristics 96 (24.4 %) of the participant reported a previous history of depression and 21 (5.3 %) of the women reported presence of history of depression in the family. Among the respondents 4 (1 %) had used tobacco while 99 (25.2 %) had used alcohol at least once in the last twelve months. The proportion of pregnant women who had used cannabis and khat at least once in the last twelve months were 2 (0.5 %) and 7 (1.8 %), respectively.

### Prevalence of antenatal depression

The internal consistency of EPDS tool was acceptable (Cronbach’s α =0.77). The prevalence of antenatal depression (≥13 on EPDS score) was 24.94 % (95 % CI: 20.85–29.30 %). The prevalence of antenatal depression among first trimester women was 5.1 % while it was 27.6 and 67.3 % among second and third trimester women respectively.

### Factors associated with antenatal depression (Logistic regression analysis)

The following variables were not significantly associated with antenatal depression in the bivariate analysis: Trimester, having previous pregnancy, previous pregnancy & labor complication, number of ever born children, previous history of still birth, previous history of abortion, history of chronic illness, age of the respondent and substance use in the last 12 months. As shown in Table 3 marital status, educational level, planned pregnancy, partners’ feeling about the pregnancy, fathers’ support, community support, and history of depression were statistically associated with depression in the bivariate analysis. However unplanned pregnancy, pregnant women experienced lack of baby’s father support, and previous history of depression were the associated factors with antenatal depression among pregnant women in the multivariable model.

**Table 3:** Factors associated with antenatal depression among pregnant women attending public health centers ANC clinic in Addis Ababa, April 2012. (*n* = 393)

Explanatory variables	COR (95 % CI)	AOR (95 % CI)
Current marital status		
Non married (Single, divorced and widowed)	4.734 (2.394–9.361)	1.799 (0.728–4.441)
Married	1	1
Educational status		
No formal education	3.316 (1.256–8.750)	2.46 (0.849–7.114)
Elementary school	2.625 (1.108–6.221)	2.0 (0.783–5.11)
Secondary school	2.227 (0.907–5.472)	1.652 (0.63–4.332)
College and above	1	1
Planned pregnancy		
No	3.901 (2.418–6.292)	2.779 (1.594–4.846)
Yes	1	1
Partner’s feeling on current pregnancy		
Unhappy	5.496 (3.081–9.805)	1.605 (0.708–3.642)
Happy	1	1
Baby’s fathers support		
Poor	3.680 (2.266–6.002)	1.89 (1.064–3.358)
Good	1	1
Community’s support		
Poor	2.107 (1.074–4.136)	0.819 (0.370–1.814)
Good	1	1
History of depression		
Yes	3.347 (2.036–5.503)	2.569 (1.475–4.475 )
No	1	1

As shown in Table 3 antenatal depression was significantly higher among women who had not planned their current pregnancy. Those women who had not planned their current pregnancy were 2.78 times more likely to have antenatal depression than those who had planned their pregnancy [AOR = 2.78(95 % CI: 1.59–4.85)]. Absence of support from baby’s father was also associated with higher odds of having antenatal depression. Pregnant women who experienced lack of support from the baby’s father were 89 % higher odds of having antenatal depression when compared with women who got support from the baby’s father [AOR = 1.89(95 % CI: 1.06–3.36)]. History of depression was found one of the factors associated with antenatal depression. Those pregnant women who had previous history of depression were nearly three times at higher odds of having depression as compared to pregnant women who had no history of depression [AOR = 2.57(95 % CI: 1.48–4.48 )]. Educational level, community’s support and partner’s feeling on the current pregnancy were not significantly associated with depression during pregnancy in the multivariable logistic regression model.

## Discussion

This study showed that there was a high prevalence of antenatal depression among pregnant women who have ANC follows up at public Health centers in Addis Ababa. The finding showed that 24.94 % (95 % CI: 20.85-29.30) of the antenatal care attendee pregnant women had depression based on the EDPS. Many studies reported higher prevalence of antenatal depression such as 39.5 % in Tanzania [11], 39 % in two Cape Town peri-urban settlements [12] and 56 % in Jamaica [34]. A relatively similar prevalence was reported from Brazil (20.2 %) [11] and Rural Bangladesh (18 %) [9]. The methodological differences between studies and different measurement tools might attribute to the difference in the prevalence of depression among these countries. The socio demographic and economic differences might also attribute for the difference in prevalence of antenatal depression between this study and the studies from other developing countries.

Antenatal depression was significantly higher among women who were not planned on their current pregnancy. Those women who were not planned on their current pregnancy were 2.58 times more likely to have antenatal depression than those pregnant women who had planned the pregnancy. This is possibly because pregnancy causes physical, psychological and hormonal changes [35] that is why pregnancy needs physical, psychological and financial preparation. A consistent finding was also reported from rural Southwestern Ethiopia [29]. Other supporting findings regarding the importance of planning pregnancy for prevention of antenatal depression were reported from different part of the world [13, 36–39]. Unintended pregnancy was found as risk factor perinatal mental disorders in women in low- and lower-middle-income countries [8]. However, a study done in two Cape Town peri-urban settlements did not found a significant association between unwanted pregnancy and antenatal depression [12].

Experiencing lack of baby’s father support was found to be a statistically significant factor associated with depression during pregnancy. This is possibly because those women who receive partner’s support during their pregnancy well empowered to deal with their pregnancy and their home responsibility. This finding agreed with many studies conducted in different countries [8, 9, 12, 33]. It is also possible that depressed women might feel that the support they receive is not sufficient.

A previous history of depression was a significant factor that associated with the development of antenatal depression. Those pregnant women might be more biologically vulnerable to depression, and the hormonal changes of pregnancy increase vulnerability to depression, or their psycho-social context may make them vulnerable to recurrent depression. This is also consistent with many other studies [8, 9, 12, 40, 41].

The mental health services are not adequate in Ethiopia. However this study implicates that the problem of antenatal depression is rampant even in the capital of the country. This recalled the importance of integrating mental health services with maternal health program. In the context of low- and middle-income countries, designing both treatment and preventive interventions are important to reduce the burden of mental health problem. A systematic review study concluded that in low and middle income countries, the burden of common perinatal mental disorders can be reduced through mental health interventions delivered by supervised non-specialists [42].

This study has limitations. EDPS is not a diagnostic aid, it is a screening method. Making a diagnosis of antenatal depression based on EDPS scale without psychiatric examination is difficult. Women in the sample were recruited from Public Health Centers in Addis Ababa who attended during the study period. They might not be representative of all antenatal care clients, and the sample did not include pregnant women who did not attend for antenatal care and those attended ANC at private health institutions. Majority of public health center users in Addis Ababa are people from low socioeconomic status which question the representativeness of this study across all socioeconomic groups. The other limitation is that major life events and possible co-morbid diagnosis such as HIV status, gender violence, anxiety or dysthymia were not assessed. The effect of nutritional status such as anemia was also not investigated in this study.

## Conclusion

One quarter of the pregnant women attending Addis Ababa Public Health Centers ANC clinic were depressed based on the EPDS. Further examination of these pregnant women by a psychiatrist to confirm such diagnosis is recommended. Unplanned pregnancy, experiencing lack of baby’s father support and previous history of depression were factors independently associated with antenatal depression.

Health care professionals need to enquire about the relevant risk factors as part of their overall assessment giving attention to those pregnant women who had unplanned pregnancy, who have poor partner support, and who had a previous history of depression. Promotion of family planning, male involvement in maternal health especially during pregnancy and integration of mental health service with existing maternal health care as well as strengthening the referral system among public health centers were the recommended interventions.
